# Toward Single Atom Chains with Exfoliated Tellurium

**DOI:** 10.1186/s11671-017-2255-x

**Published:** 2017-08-10

**Authors:** Hugh O. H. Churchill, Gregory J. Salamo, Shui-Qing Yu, Takayuki Hironaka, Xian Hu, Jeb Stacy, Ishiang Shih

**Affiliations:** 1 Department of Physics, University of Arkansas, Fayetteville, AR 72701 USA; 2 Institute for Nanoscience and Engineering, University of Arkansas, Fayetteville, AR 72701 USA; 3 Department of Electrical Engineering, University of Arkansas, Fayetteville, AR 72701 USA; 4 Department of Electrical and Computer Engineering, McGill University, Montreal, QC H3A 0G4 Canada

**Keywords:** Atom chain, Tellurium, Exfoliation, 1D layered material

## Abstract

We demonstrate that the atom chain structure of Te allows it to be exfoliated as ultra-thin flakes and nanowires. Atomic force microscopy of exfoliated Te shows that thicknesses of 1–2 nm and widths below 100 nm can be exfoliated with this method. The Raman modes of exfoliated Te match those of bulk Te, with a slight shift (4 cm^−1^) due to a hardening of the A_1_ and E modes. Polarized Raman spectroscopy is used to determine the crystal orientation of exfoliated Te flakes. These experiments establish exfoliation as a route to achieve nanoscale trigonal Te while also demonstrating the potential for fabrication of single atom chains of Te.

## Background

Dominated by carbon nanotubes and semiconductor nanowires, one dimensional (1D) materials have been extensively investigated for their extraordinary properties for electronics, photonics, and optoelectronics [1, 2]. Opportunities provided by 1D materials include transistors scaled to the smallest possible dimensions [3, 4], extremely sensitive chemical and biological sensors [5, 6], and unique electronic phenomena originating from the similarity of optical fibers and ballistic electrons inside a 1D wire [7, 8]. Progress with carbon nanotubes for most applications has been hampered by chirality randomness, and at the smallest diameters, semiconductor nanowire properties are degraded by surface dangling bonds. Consequently, the focus of low-dimensional material research has shifted primarily to two-dimensional (2D) layered materials, which combine atomic-scale thickness and high-performance physical properties by virtue of weak bonding in one direction [9–13].

The layered material concept may be generalized from 2D materials, with weak bonds in one direction, to 1D materials, with weak bonds in two directions. Many 1D weakly bonded solids are now known [14, 15]. One-dimensional weakly bonded materials may be separated to produce small diameter nanowires, as has been done with Li_2_Mo_6_Se_6_ [16, 17]. We argue that 1D weakly bonded materials present an opportunity to revisit 1D materials, with a new possibility to achieve single atom chains with atomic-scale diameters and an expectation of new physical properties stemming from crystal structures that are distinct from both carbon nanotubes and semiconductor nanowires. The anisotropic structure of 1D weakly bonded materials may allow single atom chains to be created by exfoliation, or possibly directly grown by molecular beam epitaxy or chemical vapor deposition.

**Fig. 1:**
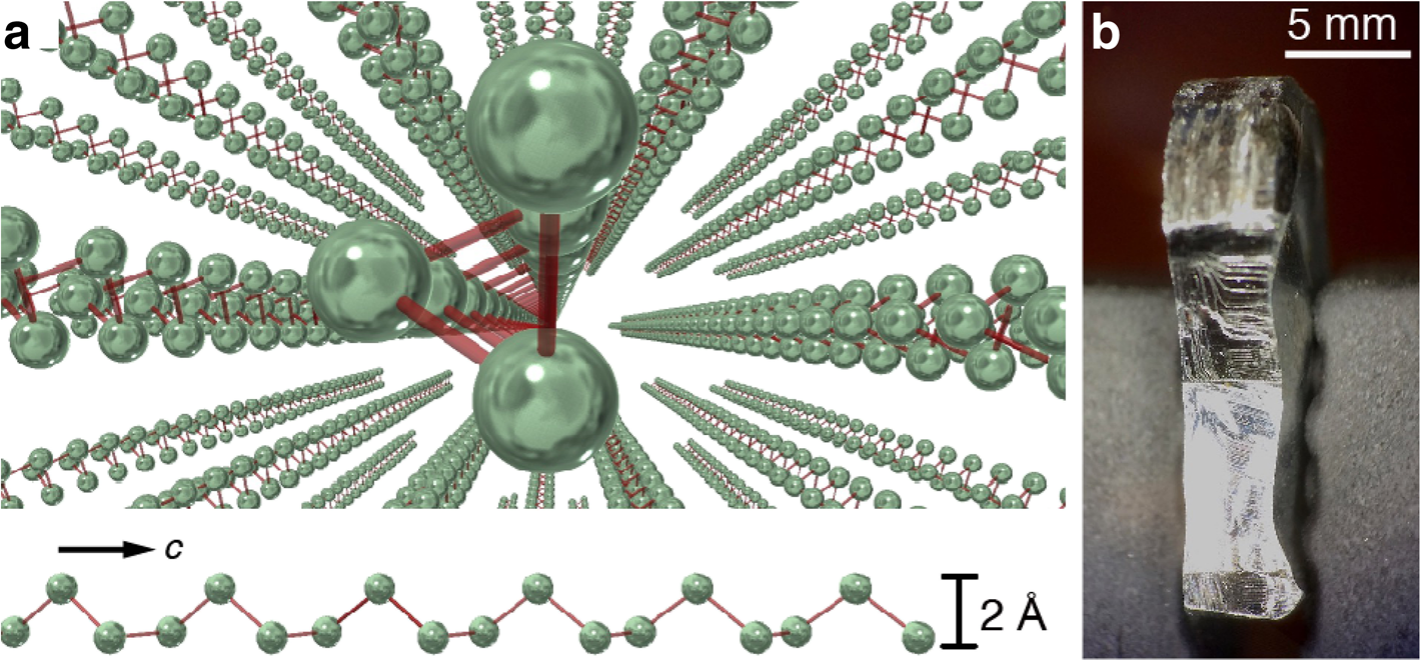
**a** Schematic of Te single crystal formed by single atom chains bonded by van der Waals force (*top*) and side view of Te chain structure (*bottom*). Note: 2 Å is the height of the triangular cross-section of a chain while the inter-chain distance is 3.4 Å. **b** Te single crystal used for exfoliation

Two exemplary 1D weakly bonded materials are trigonal Se and Te, which have lattices consisting of spiral chains oriented along the *c*-axis, each spiral having three atoms per turn with adjacent chains arranged hexagonally (Fig. 1). The chains are bound together to form a single crystal through the van der Waals force [18] or perhaps more accurately as a weakly bonded solid [19]. In this letter, we report mechanical exfoliation of trigonal Te single crystals to obtain nanoscale Te flakes and wires, which demonstrate the potential for fabrication of single atom chains and a new platform for 1D electronics and photonics.

While there are many 1D weakly bonded materials from which to choose, several properties of isolated Se and Te semiconductor atom chains set them apart from other 1D atomic layered materials. For example:

1. They are predicted to have direct semiconducting band gaps of 1 and 2 eV for Te and Se, respectively, with strongly thickness-dependent band gaps [19], creating new opportunities for tiny, wavelength-tunable detectors and emitters.
2. The helical structure of Se and Te chains is expected to confer unique electrical, optical, and mechanical properties, including novel spin-orbit coupling effects boosted by heavy Se and Te atoms [20], negative compressibility and band gap narrowing under pressure and strain [21], and extraordinary flexibility greater than typical elastic polymers [22].
3. Since they are composed of a single element, an isolated Se or Te atom chain would have the smallest diameter of any known 1D material. The height of the triangular spiral cross-section is 2 Å, and the inter-chain distance is 3.4 Å [23].

Experimental demonstration of the atom chain concept originates with STM manipulation of individual atoms on a substrate to achieve linear and planar arrays of coupled atoms [24, 25]. In addition to atom-by-atom assembly on surfaces, step edges of substrates have been decorated with atom chains [26], and self-assembled growth has been used to create large-area arrays of atom chains [27]. However, depending on the approach, all these pioneering experiments do not allow 1D structures to be created over large scales, choice of materials is limited, or the structure is strongly bound to the substrate. In principle atom chains derived from 1D weakly bonded materials could overcome these limitations.

To date, the anisotropic structure of Se and Te has permitted growth of small diameter nanowires [28, 29], self-assembly of single chains inside zeolite pores [30, 31] and carbon nanotubes [32], the growth of 2D monolayer trigonal Te on graphene [33], and solution-growth of 2D Te [34, 35]. This earlier work demonstrates the tendency of Te to form chains and nanowires that are relatively stable mechanically and chemically outside the bulk Te crystal structure. Our objective is to use exfoliation of solid Te as a route to obtain single atom chains.

## Methods

**Fig. 2:**
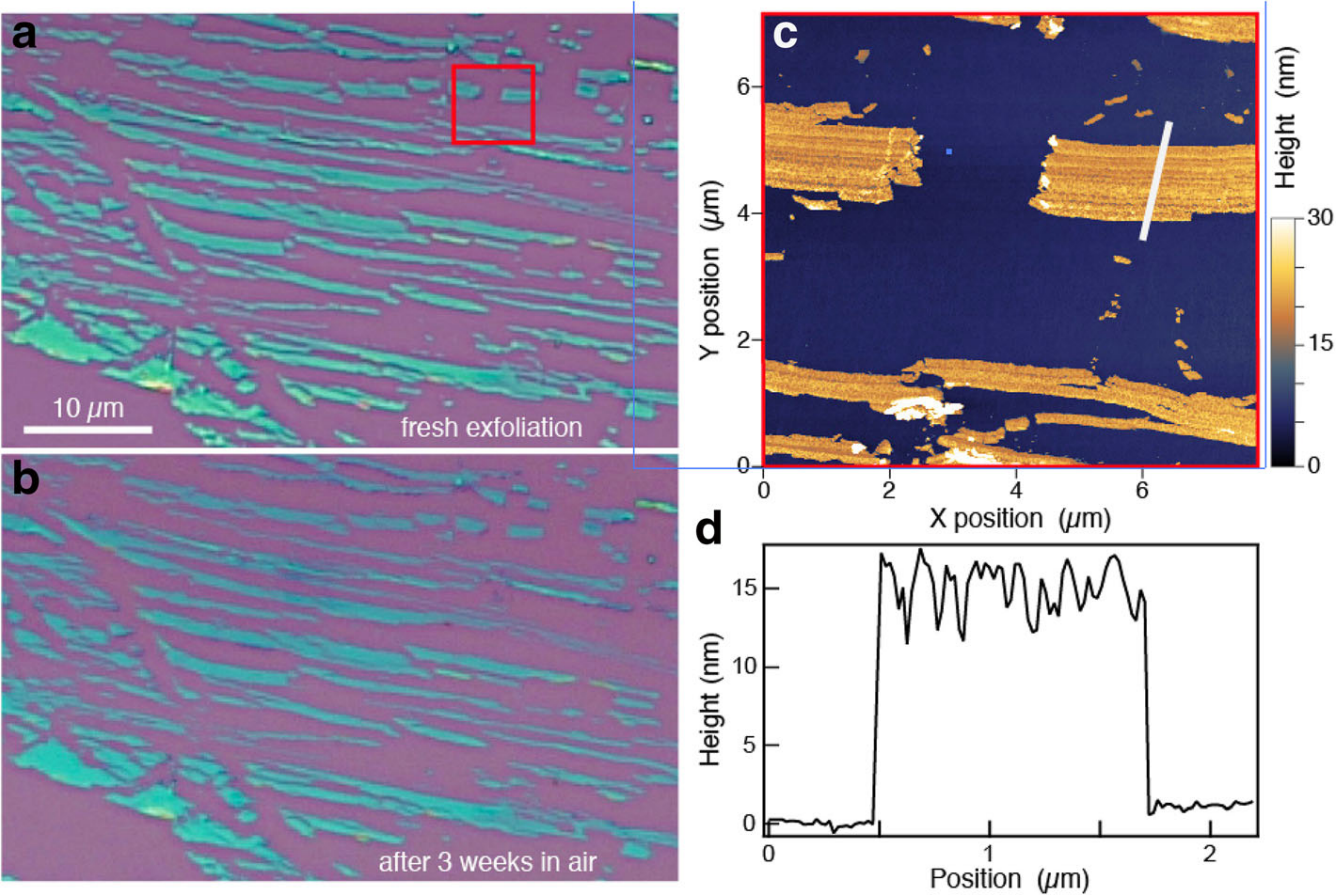
**a** Te exfoliated on a Si/SiO2 substrate, imaged immediately after exfoliation. **b** The same sample as in (**a**) after storage in air for 3 weeks. **c** AFM height image of the area inside the *red square* in (**a**). **d** Height profile along the *white line* shown in (**c**)

To provide evidence for the potential for fabrication of single atom chains, we investigated Te rather than Se because of the availability of large, high-quality Te single crystals [36]. Prior to exfoliation, silicon substrates with 90 or 300 nm of thermal oxide were sonicated in acetone and isopropanol, then treated with oxygen plasma to improve adhesion of Te. Trigonal Te single crystals were mechanically exfoliated, without tape, directly on the silicon substrates [37] by manually sliding a freshly cleaved facet of Te on the substrate. We obtained the best results with the *c*-axis perpendicular to the direction of motion. For Te exfoliation, we have found this method to be significantly superior to tape exfoliation, which likely reflects an important difference in the bonding between 1D and 2D layered materials. Thin Te flakes were identified by contrast in an optical microscope (Fig. 2a). Thin Te flakes show up with a progression of colors in reflected light microscopy with the thinnest crystals appearing as darker greens and blues on this silicon substrate.

## Results and Discussion

Tellurium was exfoliated in anisotropic linear bands with lengths up to 50 μm (Fig. 2a). Atomic force microscopy of some of these bands reveals heights in the 10–15 nm range (Fig. 2c), with ridges running along the length of the bands that are evident in both the height image and a height profile taken perpendicular to one of the bands as shown in Fig. 2d. The modulated surface pattern and variation in wire width are evidence that the atom chains randomly break away from the bulk crystal both laterally and vertically, unlike 2D layered materials such as graphene which exfoliate with mostly flat surfaces whether a tape or sliding technique is used. We were able to obtain wires of 1–2 nm thickness using this sliding technique.

**Fig. 3:**
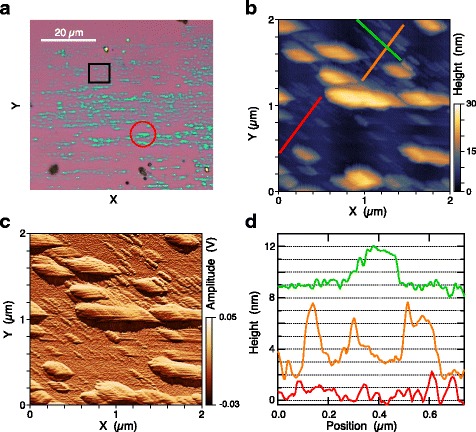
**a** Optical micrograph of a second exfoliated Te sample. The *red circle* indicates the region used for Raman spectroscopy. **b** AFM height and (**c**) tapping mode amplitude images of the region indicated by the *black square* in (**a**). **d** Height profiles along the *red*, *orange*, and *green lines* in (**b**), perpendicular to the *c-*axis direction for *red* and *orange*, parallel for *green*. The *orange* and *green* profiles are offset vertically for clarity

For example, the atomic force images of the second sample reveal a similar anisotropic structure of the exfoliated material (Fig. 3a), as well as significantly narrower Te nanowires with heights in the subnanometer range (Fig. 3b–d) or at least corresponding to two to four chains for an inter-chain distance of 3.4 Å [23]. These ultrathin Te nanowires have lengths of 100–200 nm (Fig. 3a). A height profile taken along the *c*-axis direction (green line in Fig. 3b, green curve in Fig. 3d) indicates that the surface roughness along the top of this 2–3-nm tall nanowire is comparable to or less than that of the SiO_2_ substrate.

Stability in ambient environment is a concern for any newly exfoliated material because surface reactions that are negligible in bulk materials can dominate the properties of ultrathin exfoliated materials. An optical image of the same Te sample in Fig. 2a is shown in Fig. 2b after storage for 3 weeks in air. Aside from differences in color contrast due to camera settings, the aged sample appears virtually the same as when it was freshly exfoliated. In particular, we note a complete absence of the blistering that occurs when 2D black phosphorus degrades in air [38]. This observation is consistent with the observation that the timescale for degradation of Te nanowires in various solvents such as water is not indefinite but quite long, from hours to days [39].

**Fig. 4:**
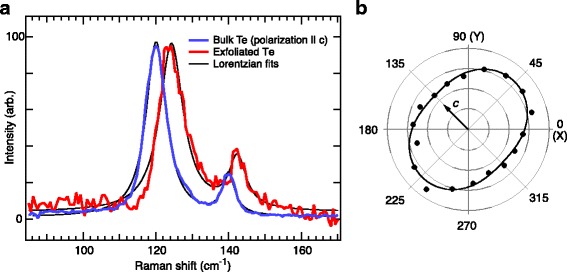
**a** Raman scattering spectrum of bulk Te crystal (*blue*) and an exfoliated flake (*red*), under the same excitation conditions (633 nm, polarization parallel to *c*-axis). Spectra are normalized to the height of the dominant A1 peak. Fits (*black curves*) are a sum of two Lorentzians. **b** Polar plot of Raman intensity averaged over the spectral range in (**a**) as a function of linear excitation polarization angle relative to the *c*-axis (plot origin is zero intensity). The fit is a sine function plus a constant. The *black arrow* indicates the *c*-axis direction (see text)

We further characterize the exfoliated Te by Raman spectroscopy. The Raman spectrum of bulk Te at room temperature is dominated by two sets of modes: an A_1_ singlet at 120 cm^−1^ and a pair of E doublets at 92 (104) and 141 (141) for transverse (longitudinal) phonons [40]. The A_1_ and E modes of trigonal Te may be visualized as symmetric and antisymmetric breathing modes of the triangular cross-section of the Te chain [41]. This spectrum is reproduced in Fig. 4a for an excitation wavelength of 633 nm, with the lower E mode absent because of the polarization direction of the incident light [42]. Peak positions agree with those reported in Ref. [40] to better than 1 cm^−1^. We note that excitation at 633 nm is near a resonance with the dielectric function of bulk Te; off-resonant excitation at 532 nm produces significantly less Raman scattering intensity [43].

The Raman spectrum of an approximately 30-nm-thick Te flake (red circle in Fig. 3a) shows the same two peaks, shifted to slightly higher frequencies (Fig. 4a). The measured Raman peak of the silicon substrate at 520.9 cm^−1^ (not shown) indicates that the spectrometer is calibrated to better than 1 cm^−1^. We also note that the exfoliated Te spectrum shown in Fig. 4a, which was measured in air several weeks after exfoliation, is not consistent with the Raman spectra of either amorphous [44] or oxidized Te [45], which also establishes the environmental stability of ultrathin exfoliated Te. Despite a slight asymmetry in the Raman peaks for both bulk and exfoliated Te, a pair of Lorentzians fits the spectra reasonably well (black curves in Fig. 4a). Peak parameters extracted from the fits indicate a mode hardening for the exfoliated flake relative to the bulk crystal of 4 cm^−1^ for the A_1_ mode and 2 cm^−1^ for the E mode.

One interpretation of this mode hardening is a flake-substrate interaction, for example, if the Te is strained as it is exfoliated on the SiO_2_ substrate. Interaction with the substrate also generically hardens the radial breathing modes of carbon nanotubes [46]. Another possibility is that inter-chain interactions are reduced in ultrathin Te because a significant fraction of chains is missing one or more neighbors. A naïve expectation would be that weaker inter-chain coupling would soften the A_1_ mode; however, applying pressure to Te crystals is known to reduce the A_1_ frequency [47]. Further, the A_1_ frequency of isolated Te chains inside zeolite nanopores, where inter-chain coupling is zero (or significantly less than for bulk, considering the 6.6 Å nanopore diameter), is much higher than in bulk Te at 172 cm^−1^ [48]. The observation that reduced inter-chain coupling hardens Te Raman modes is explained by a competition between inter- and intra-chain forces in Ref. [23]. Our measurement of a smaller shift for the E mode than the A_1_ mode (Fig. 4a) is also consistent with the pressure dependence reported in Ref. [43], but substrate-induced strain may be expected to produce similar behavior. We are unable to conclude within the scope of this work whether substrate interaction or reduced inter-chain interactions are responsible for the spectral shifts we observe.

For the sample shown in Fig. 3, both optical and atomic force microscopy display elongated, horizontally aligned Te flakes, which suggests that the *c*-axis of the Te crystal is horizontal in these images. However, the AFM images (Fig. 3b, c) also show that a significant fraction of the exfoliated flakes, particularly the thinnest ones, are tilted 45° away from horizontal. To confirm the crystal orientation of this sample, we use polarization-resolved Raman spectroscopy. The polarization of the excitation beam was rotated with a half-wave plate, and the integrated Raman intensity from 85 to 170 cm^−1^ is shown in Fig. 4b. The intensities were normalized by the laser power under the microscope objective measured at each polarization angle. The Raman intensity shows two maxima within one full rotation, located at 45° and 225° with respect to the *X* and *Y* axes defined in the microscope images (Fig. 3). The intensity varies approximately sinusoidal (black curve in Fig. 4b), with an amplitude of +/−15% over a constant background.

Meanwhile, the optical absorption of bulk Te at 633 nm is stronger for light polarized perpendicular to the *c-*axis than for parallel polarization [49]. For Te flakes with nearly bulk-like optical properties (Fig. 4a), we therefore expect Raman intensity to be higher for light polarized perpendicular to the *c*-axis. Based on the angle of the Raman maximum in Fig. 4b, we conclude that the Te nanowires oriented at 45° in Fig. 3b, c are elongated parallel to the *c*-axis for that sample. Because different Te flakes on the same substrate were used for Raman spectroscopy and AFM, an assumption of this conclusion is that the crystal axes are the same for all exfoliated flakes shown in Fig. 3a. This assumption would not be appropriate for flakes prepared by the traditional tape exfoliation method, but it is a reasonable assumption for the unidirectional rubbing technique used here. These observations demonstrate that polarized Raman spectroscopy is sufficient to determine the crystal orientation of nanoscale exfoliated Te. This technique is useful in practice given that optical and atomic force microscopy do not provide unambiguous information about crystal orientation. As the thickness and width of exfoliated Te approaches the single atom chain limit, we expect a cross-over in the crystal direction associated with maximum Raman scattering because isolated Te chains inside nanopores have maximum Raman intensity for polarization parallel to the *c*-axis [48].

## Conclusions

We have introduced trigonal Te as a weakly bonded material capable of being exfoliated to produce ultrathin Te single crystals. We demonstrate that the atom chain structure of Te allows it to be exfoliated as two-dimensional flakes and one-dimensional nanowires. Atomic force microscopy of exfoliated Te shows that thicknesses of 1–2 nm and wires of about 100 nm width can be exfoliated with this method. The Raman modes of exfoliated Te match those of bulk Te, with a slight shift (4 cm^−1^) due to a hardening of the A_1_ and E modes. Polarized Raman spectroscopy is used to determine the crystal orientation of exfoliated Te flakes. These experiments establish exfoliation as a route to achieve nanoscale trigonal Te while demonstrating the potential for fabrication of single atom chains of Te. Our current efforts are focused on producing Te or Se single atom chains by molecular beam epitaxy or by improving exfoliation.
